# Visual stimulation by extensive visual media consumption can be beneficial for motor learning

**DOI:** 10.1038/s41598-023-49415-4

**Published:** 2023-12-12

**Authors:** Matthias Nuernberger, Kathrin Finke, Lisa Nuernberger, Adriana L. Ruiz-Rizzo, Christian Gaser, Carsten Klingner, Otto W. Witte, Stefan Brodoehl

**Affiliations:** 1 Department of Neurology, Jena University Hospital, Friedrich Schiller University, Jena, Germany; 2 Biomagnetic Center, Department of Neurology, Jena University Hospital, Friedrich Schiller University, Jena, Germany; 3 German Center for Mental Health (DZPG), Department of Psychiatry and Psychotherapy, Jena University Hospital, Jena, Germany

**Keywords:** Neuroscience, Motor control, Sensorimotor processing, Visual system

## Abstract

In this randomized controlled intervention trial, we investigated whether intense visual stimulation through television watching can enhance visual information processing and motor learning performance. 74 healthy young adults were trained in a motor skill with visual information processing demands while being accommodated in a controlled environment for five days. The experimental manipulation (*n* = 37) consisted of prolonged television watching (i.e., 8 h/day, + 62.5% on average) to induce intense exposure to visual stimulation. The control group (*n* = 37) did not consume visual media. The groups were compared by motor learning performance throughout the study as well as pre/post visual attention parameters and resting-state network connectivity in functional MRI. We found that the intervention group performed significantly better in the motor learning task (+ 8.21% (95%-CI[12.04, 4.31], t(70) = 4.23, p < 0.001) while showing an increased capacity of visual short-term memory (+ 0.254, *t*(58) = − 3.19, *p* = 0.002) and increased connectivity between visual and motor-learning associated resting-state networks. Our findings suggest that the human brain might enter a state of accentuated visuomotor integration to support the implementation of motor learning with visual information processing demands if challenged by ample input of visual stimulation. Further investigation is needed to evaluate the persistence of this effect regarding participants exposed to accustomed amounts of visual media consumption.

Clinical Trials Registration: This trial was registered in the German Clinical Trials Register/Deutsches Register klinischer Studien (DRKS): DRKS00019955.

## Introduction

In 2020 an adult between 18 and 90 years living in central Europe spent 220 min on average per day watching television^1–3^. Approximately the same amount was reached in 2019, prior to the outbreak of the Covid19 pandemic. This makes watching television the most common leisure time occupation. While extensive research is conducted concerning this matter, many aspects are still unknown. Does watching television affect our cognitive abilities? Does it alter our brain function? We know that visual information from our environment is processed in a privileged manner in comparison to other senses (“Colavita visual dominance effect”)^4^. This can also be observed in the McGurk- or Ventriloquist-effect^5,6^, where non-visual sensory input is omitted for the sake of visual information. A large part of our daily visual information input is provided by visual media consumption. It is known that video games can alter visual information processing^7–9^. Watching television can be considered a passive variation of playing video games because of the equally high visual information input. Whilst the passive aspect is accompanied by adverse effects on our body (e.g., reduced muscle mass through inactivity, elevated risk for obesity, and diminished cardiorespiratory function^10–13^), the benefit for visual information processing might be conserved. On the other hand, retrospective analyses have revealed a negative impact of visual media-consumption on the developing brain of children, resulting in diminished cognitive abilities^14,15^ and behavioural disorders^16,17^. The adult brain might be confronted with the same unfavourable effects, for which there are indications especially above 210 min of watching television per day^18^. Yet television can improve daily structure and social integration for elderly by supporting daily routine^19^. Furthermore, the “social surrogacy hypothesis” suggests that television can also provide emotional support for people suffering from social stress^20^.

However, most of these assumptions are not based on robust data and are discussed controversially^21^. Reliable data is sparse and there are few prospective trials in this area of interest. Especially the aspect of motor learning has been left almost untouched. Motor learning commonly means to obtain new and skilled movements, primarily in form of a sequence of movement patterns^22^. It might be expected that there is an influence on motor skills and their acquisition because they are a central element in our cognitive processes. The established “Model of metamodal cortex”^23^ by Pascual-Leone and Hamilton describes that cerebral cortex function can change depending on the presently needed processing mode and effort regardless of its primary area of activity. Physiological adaptations in brain activities could then be reflected in changes in information exchange between cortical networks^24^ or grey matter volume^25^. It is possible that intense visual stimulation by television is seizing up cerebral resources which otherwise would be employed for example in motor learning or somatosensory perception. On the other hand, it is imaginable that intense visual stimulation is recruiting additional cerebral resources which subsequently facilitate progress in cognitive tasks like motor learning. This might be due to the fact that television presents artificial (audio-)visual information input, which may be scalable but will in most cases differ from real experiences and tend to offer a higher information load: a movie for example contains sequences of different scenes and settings populated by different people or showing different events. Real experiences however are not only often influenceable by the individual but also slower and in general more predictable, lowering the information load. Integrating the “Model of metamodal cortex” and metamodal processing, a measurable effect on cognitive information processing domains could be expected.

### Hypothesis

We hypothesize that intense visual stimulation via extensive consumption of visual media enhances visual information processing and improves the acquisition of a motor skill with visual information processing demands. We suggest that these effects will be accompanied by increased functional connectivity between brain networks involved in visual information processing and motor learning, increased grey matter volume in these areas and diminished somatosensory perception capabilities.

We tested this hypothesis on the experimental group by inducing intense visual stimulation through long daily television watching in a controlled environment. We investigated whether this would boost motor learning performance (represented by touch-typing on the PC keyboard), increase visual processing speed as well as visual short-term memory capacity as described in the Theory of Visual Attention (TVA)^26^, enhance functional connectivity (FC) between resting-state brain networks (RSN) in functional MRI (fMRI) involved in visuomotor learning, and enlarge grey matter volume in these areas measured by Voxel-based morphometry (VBM)^27^. Furthermore, we looked for diminished somatosensory perception capabilities in Grating-orientation task (GOT) and Mechanical-detection threshold (MDT) as a compensational mechanism. To control for retest effects and nonspecific factors (e.g., social interactions), we included a control group, which was accommodated in the same controlled environment for motor skill training but did not watch television and was limited to non-screen-based leisure time activities like reading.

## Results

Our study was a randomized controlled intervention trial. Subjects were randomized in one of two groups: the experimental group (TV, n = 37) watched a minimum of 8 h of visual media like television per day. The control group (NOTV, n = 37) did not consume any visual media. Both groups were accommodated in a controlled environment for 5 days and meanwhile completed a course in touch-typing on the PC keyboard. Touch-typing performance served as motor learning surrogate, as this skill was new to all participants. Before and after the experiment we conducted assessments (pre & post) on resting-state functional connectivity (fMRI), grey matter volume (VBM), visual attention (TVA), and somatosensory perception (MDT & GOT).

### Touch-typing

#### Baseline

**Figure 1:**
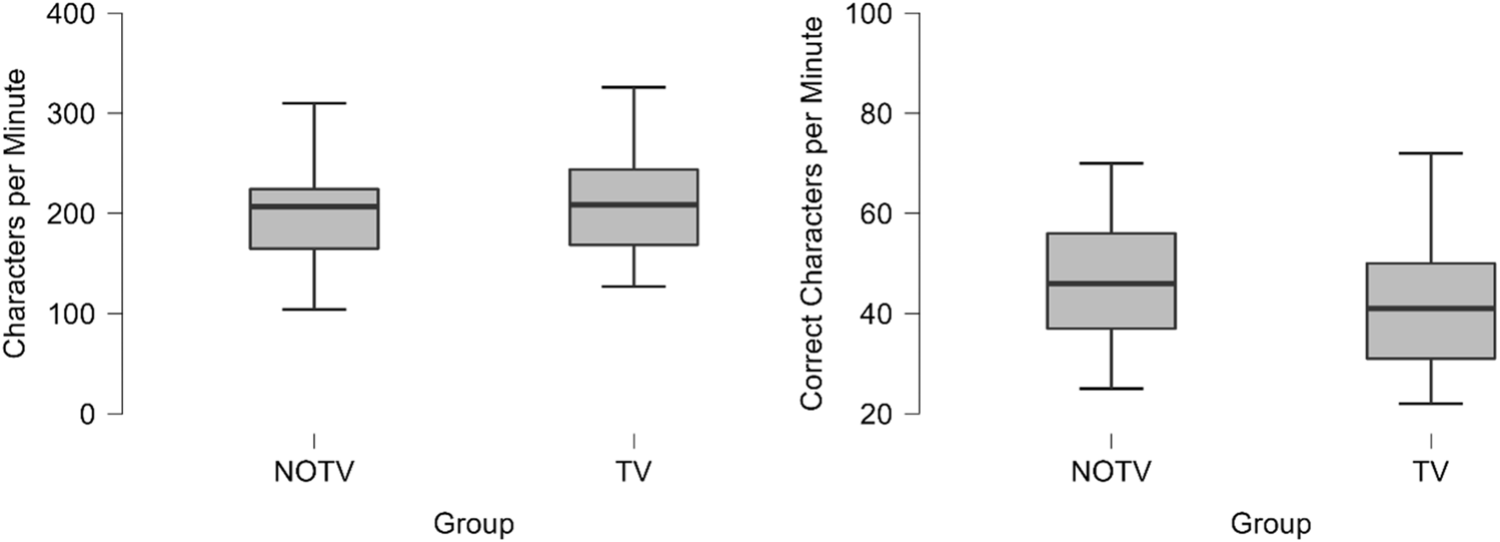
Performance in touch-typing at baseline (dictation on initial assesment and first training session). Note: Baseline in touch-typing skill. Left: Total characters per minute in a 10-min dictation at initial assessment. There was no significant difference between the groups, *p* = 0.392. Right: Correct entered characters per minute in the first 45-min training session on day 1 of the trial. There was no significant difference between the groups, *p* = 0.511. Asterisk symbol (*) markes statistically significant differences between the groups.

At baseline the two groups did not differ in typing skills (see Table 5 in the supplementary material and Fig. 1), verified through comparison of the typed characters in the first training session on day 1 (*t*(70) = 0.861, *p* = 0.392) and the first dictation on initial assessment (*t*(70) = − 0.660, *p* = 0.511).

#### Touch-typing motor learning performance

##### GainL3

TV achieved a significantly higher Gain_L3_ compared to NOTV on all 5 days of the trial. More specifically, Gain_L3_ on day 2 was 12.47% higher in TV (mean between-group difference: 0.164, 95%-CI[− 0.25, 0.08], *t*(70) = 3.71, *p* < 0.001, *d* = − 0.876). 11.60% higher in TV on day 3 (mean between-group difference: 0.165, 95%-CI[− 0.27, 0.06], *t*(70) = 3.11, *p* = 0.003, *d* = − 0.734). 13.20% higher in TV on day 4 (mean between-group difference: 0.201, 95%-CI[− 0.33, 0.07], *t*(70) = 3.07, *p* = 0.003, *d* = − -0.724). 13.76% higher in TV on day 5 (mean between-group difference: 0.217, 95%-CI[− 0.36, 0.07], *t*(70) = 3.00, *p* = 0.004, *d* = − 0.708, see Fig. 8 in supplementary material).

**Figure 2:**
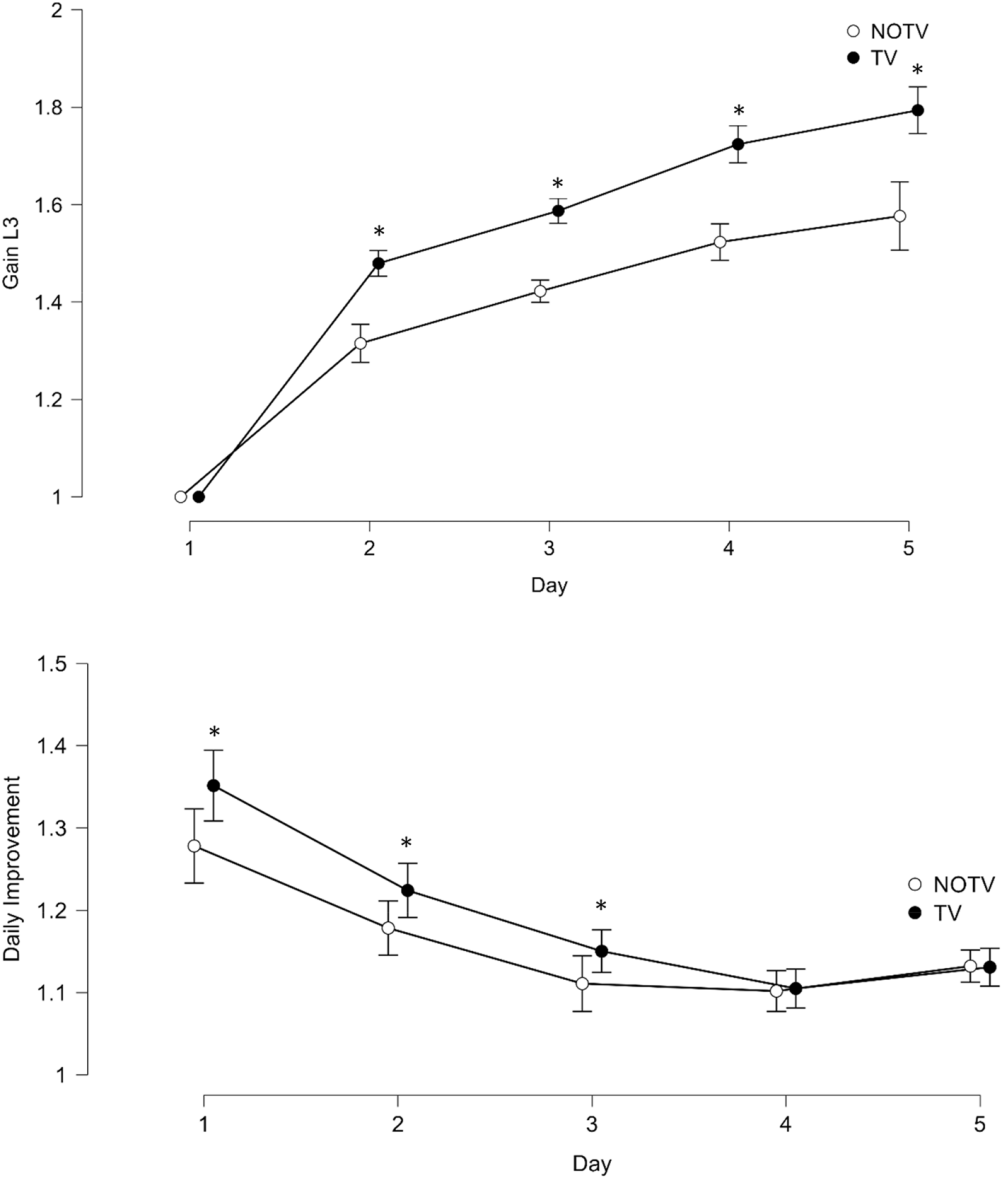
Increase in touch-typing performance—GainL3 & DI. Note: (**A**) TV showed a significantly higher improvement in correctly entered characters in the marker lesson (Gain_L3_) across all 5 days of the study. Since Gain_L3_ on day 1 served as baseline a difference on day 1 was not possible. Post-hoc analysis revealed a mean difference of 0.149 between the groups, 95%-CI[0.24, 0.06]. (**B**) TV showed a significantly higher daily improvement (DI) in total correctly entered characters on day 1, 2 and 3. On day 4 and 5 the difference was not significantly different. Post-hoc analysis revealed a significant mean difference of 0.032 between the groups, 95%-CI[− 0.06, − 0.01]. Asterisk symbol (*) markes statistically significant differences between the groups.

Mean performance levels showed a statistically significant higher Gain_L3_ in TV across all 5 days (repeated measures mixed ANOVA, *F*(1.91, 133.88) = 258.53, *p* < 0.001, η^2^ = 0.507, see Fig. 2 and Table 6 in the supplementary material). Time (i.e., day) *×* group interaction showed a different gain across different days between the groups (*F*(1.91, 133.88) = 6.617, *p* = 0.002), yet the effect size was low (η^2^ = 0.013). Tukey-adjusted post-hoc analysis revealed a mean difference of 0.149 (95%-CI[0.24, 0.06]) between the groups (*F*(1, 70) = 11.46, *p* = 0.002, η^2^ = 0.048). Further Tukey-adjusted post-hoc analysis showed that the continuous increase in Gain_L3_ was significantly different between all consecutive days in both groups except from day 4 to 5, where both groups did not increase significantly, *p* > 0.05.

##### Daily improvement (DI)

 TV achieved a significantly higher improvement by 5,84% on day 1 compared to NOTV (mean difference: 0.075, 95%-CI[− 0.15, 0.00], *t*(70) = 2.26, *p* = 0.027, *d* = 0.533). On day 2 TV improved significantly higher by 4.00% (mean difference: 0.046, 95%-CI[− 0.12, 0.03], *t*(69) = 2.06, *p* = 0.043, *d* = − 0.489). TV also achieved a significantly higher improvement by 3.60% on day 3 (mean difference: 0.039, 95%-CI[− 0.11, 0.04], *t*(70) = 2.07, *p* = 0.042, *d* = − 0.488). On day 4 there was no statistically significant difference in daily improvement between groups (+ 1.10%, mean difference: 0.012, 95%-CI[− 0.08, 0.07], *t*(71) = 0.88, *p* = 0.380). On day 5 there was no statistically significant difference in daily improvement between groups (mean difference: 0.01, 95%-CI[− 0.07, 0.08], *t*(70) = 0.74, *p* > 0.05).

A repeated measures mixed ANOVA with Greenhouse–Geisser correction and Tukey-adjusted post-hoc analysis revealed a significant mean difference of 0.032 (95%-CI[− 0.06, − 0.01]) between the groups (*F*(1, 64) = 7.74, *p* = 0.007, η^2^ = 0.018, see Fig. 2). Tukey-adjusted post-hoc analysis showed that DI of both groups was statistically different between day 1 and 2 as well as 2 and 3, *p* < 0.05. However, DI did not differ between day 3 and 4 or 4 and 5, *p* > 0.05. Therefore, there was no significant group *x* time interaction, *p* = 0.114.

##### Motor learning efficiency (MLE)

**Figure 3:**
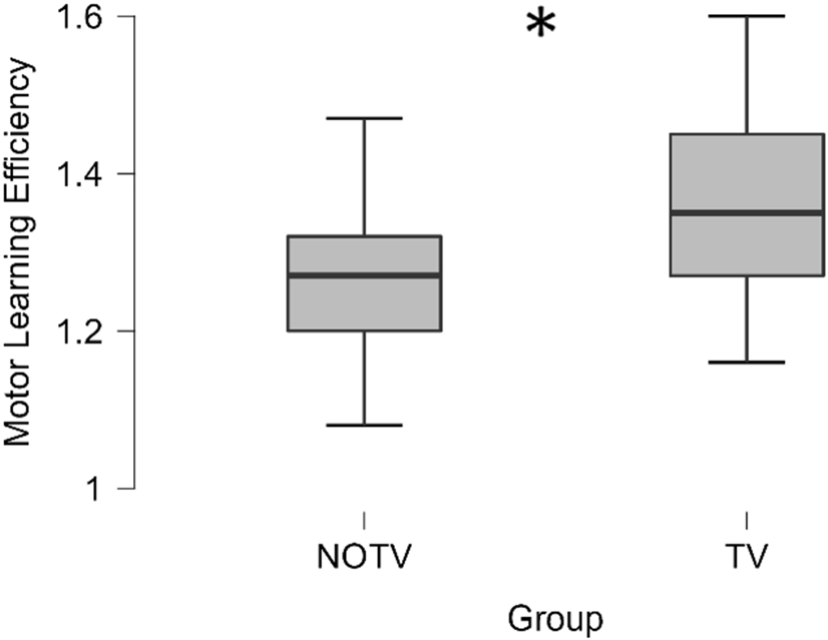
Increase in touch typing performance—motor learning efficiency (MLE). Note: MLE was significantly higher in TV (+ 8.21%) compared to NOTV, mean difference: 0.103, 95%-CI[− 0.15, 0.05], *t*(70) = 4.23, *p* < 0.001, *d* = − 0.997. Asterisk symbol (*) markes statistically significant differences between the groups.

 MLE of TV was + 8.21% (95%-CI[12.04, 4.31]) higher than in NOTV (mean difference: − 0.103, 95%-CI[− 0.15, − 0.05], t(70) = 4.23, p < 0.001, d = − 0.997, see Fig. 3).

#### Touch-typing—subgroup analysis

Gain_L3_, DI and MLE did not correlate with age, *p* > 0.05, mean age: 23, (95%-CI[22.31–23.49], SD 2.49). Gain_L3_, DI and MLE did not differ between gender or group/gender-combinations, *p* > 0.05. We found no evidence for an influence of pre-trial media consumption intensity on MLE, *p* > 0.05. There was no significant difference between three groups classified according to preferred television content during the study (Relax, Mixed, Thrill), *p* > 0.05.

### Resting-state network and task-related FC in fMRI

The subsequent classification of resting-state networks (RSN) follows the 17N parcellation of Yeo^28^ (see Table 4 in the supplementary material). Additional data is available in Table 7 in the supplementary material, Table 4 in the supplementary material. This parcellation was also used for the task-related FC during Manual Sequence Task (MST).

#### Baseline

At baseline there was no significant difference between the groups regarding FC between and within RSN, *p-FDR* > 0.01.

At baseline there was no significant difference between the groups regarding FC between and within functional networks during MST, *p-FDR* > 0.01.

At baseline there was no significant change to RSN FC after MST between the groups, *p-FDR* > 0.01.

#### Effect of Treatment (group) x Time (pre/post) interaction during Rest

Comparing RSN FC (see Table 7 in the supplementary material) between the groups before and after the experiment, the Ventral Attention Network 1 (VAN-1, right precentral area) showed a statistically significant anticorrelated change in its connectivity to Default Mode Network 1 (DMN-1, right medial prefrontal cortex): While the FC was increased in TV, it decreased in NOTV (*t* = 4.65, *p*-FDR = 0.002, *beta* = 0.19). While both groups showed a negative FC at baseline, FC in TV became positive after the intervention.

**Figure 4:**
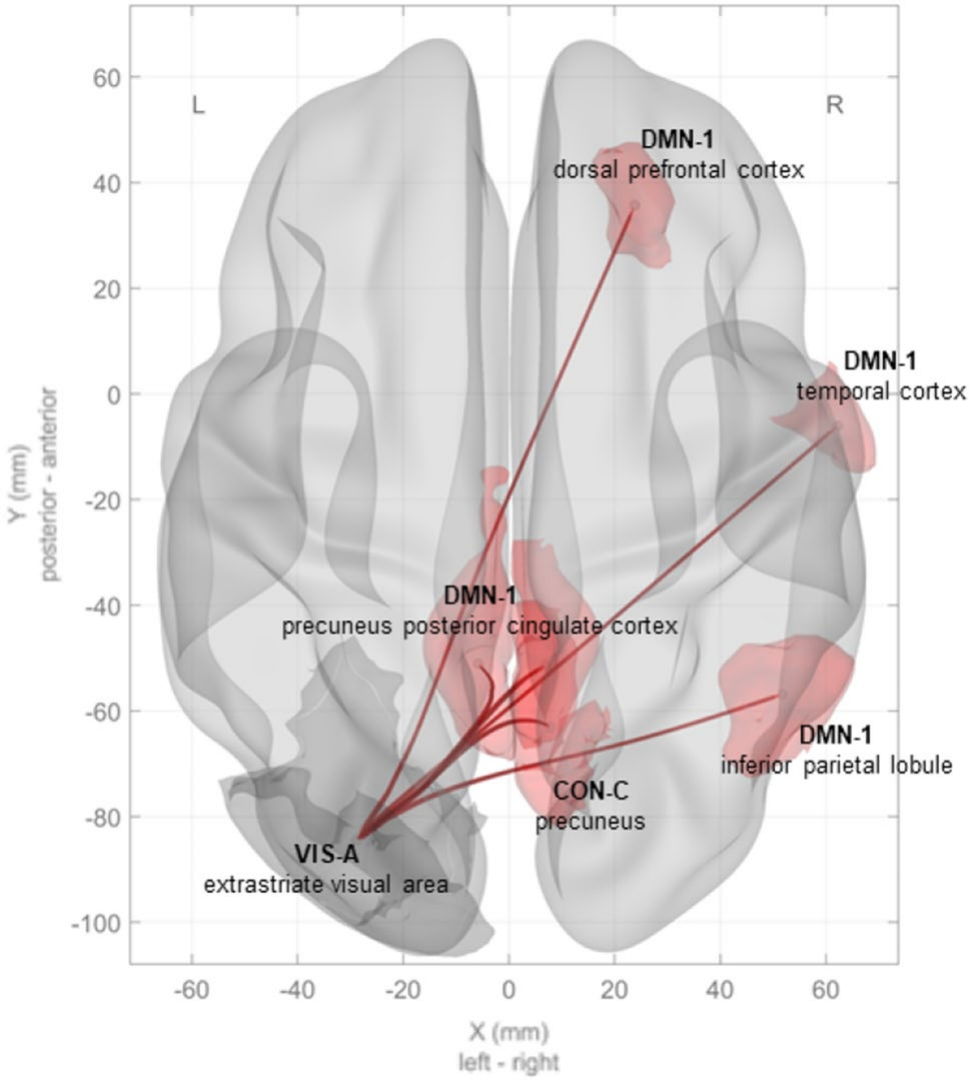
Main effect of treatment × time interaction during rest. Note: The depiction illustrates the main effect of treatment (group) and time (pre/post) interaction on RSN FC. Red lines indicate significantly increased FC between RSN, *p-FDR* < 0.05. Further statistical data is available in Table 7 in the supplementary material. VIS-A, visual Network A; DMN-1, default mode network 1; CON-C, control network C.

Furthermore, FC of the Visual Network A (VIS-A, extrastriate cortex) to DMN-1 and Control Network C (CON-C) changed distinctly: Right VIS-A showed increased FC to DMN-1 (left precuneus posterior cingulate cortex, *t* = 3.67, *p*-FDR = 0.033, *beta* = 0.19). Left VIS-A showed increased FC to DMN-1 as well (left precuneus posterior cingulate cortex, *t* = 3.64, *p*-FDR = 0.033, *beta* = 0.2). Left VIS-A additionally showed increased FC to DMN-1 (right inferior parietal lobule, *t* = 3.72, *p*-FDR = 0.024, *beta* = 0.18). Left VIS-A showed increased FC to DMN-1 (right precuneus posterior cingulate cortex, *t* = 3.62, *p*-FDR = 0.024, *beta* = 0.20). Left VIS-A showed increased FC to DMN-1 (right temporal cortex, *t* = 3.23, *p*-FDR = 0.040, *beta* = 0.15). Left VIS-A showed increased FC to DMN-1 (right dorsal prefrontal cortex, *t* = 3.14, *p*-FDR = 0.044, *beta* = 0.18). Left VIS-A showed increased FC to CON-C (right precuneus, *t* = 3.41, *p*-FDR = 0.027, *beta* = 0.18). See Fig. 4 for a graphical depiction.

#### Effect of MLE on RSN FC

The effect of Motor Learning Efficiency (MLE) as a between-subjects factor on FC change from pre to post differs between the groups. The intrinsic FC of left Somatomotor Network B (MOT-B) increased significantly through a higher MLE in the TV group: The left insular region showed higher FC with the central area (precentral gyrus, *t*(61) = 3.32, *p*-FDR = 0.0425, *beta* = 1.33) and the postcentral area (second somatosensory cortex S2, (*t*(61) = 3.43, *p*-FDR = 0.0424, *beta* = 1.51). Furthermore, left MOT-B in TV showed a higher FC to right MOT-B (auditory cortex, *t*(61) = 3.65, *p*-FDR = 0.0425, *beta* = 1.48) and VAN-A (insular cortex, (*t*(61) = 3.40, *p*-FDR = 0.0426, *beta* = 1.62).

#### Effect of Treatment x Time interaction during MST

Comparing FC during MST between the groups before and after the experiment, the right Dorsal Attention Network 1 (DAN1, right superior parietal lobule cortex/right intraparietal sulcus (IPS)) showed a statistically significant anticorrelated change in its connectivity to right Dorsal Attention Network 2 (DAN2, right temporal occipital cortex): While the FC was increased in NOTV, it decreased in TV (*t*(31) = − 3.75, *p*-FDR = 0.0046, *beta* = − 0.18). There was a very similar positive FC in both groups at baseline.

### Voxel-based morphometry (VBM) results

Comparing regional volumes in NOTV pre and post, we found a significant increase in the left middle cingulate gyrus (value: + 6.52, cluster-size: 406, MNI-coordinates [mm]: − 9 − 21 36, *p* < *0.001 uncorr.*).

In TV there was a significant volume increase in the left entorhinal area (value: + 7.86, cluster-size: 1008, MNI-coordinates [mm]: − 21 − 2 − 20, *p* < *0.001 uncorr.*) and a decrease in the right precentral gyrus (value: − 9.22, cluster-size: 298, MNI-coordinates [mm]: 48 0 53, *p* < *0.001 uncorr.*). Furthermore, we found a volume decrease in the left angular gyrus (value: − 6.60, cluster-size: 889, MNI-coordinates [mm]: − 44 − 72 52, *p* < *0.001 uncorr.*)

### Whole and partial report based on TVA

At baseline we found no evidence for a difference between the two groups in any TVA parameter, *p* > 0.05.

**Figure 5:**
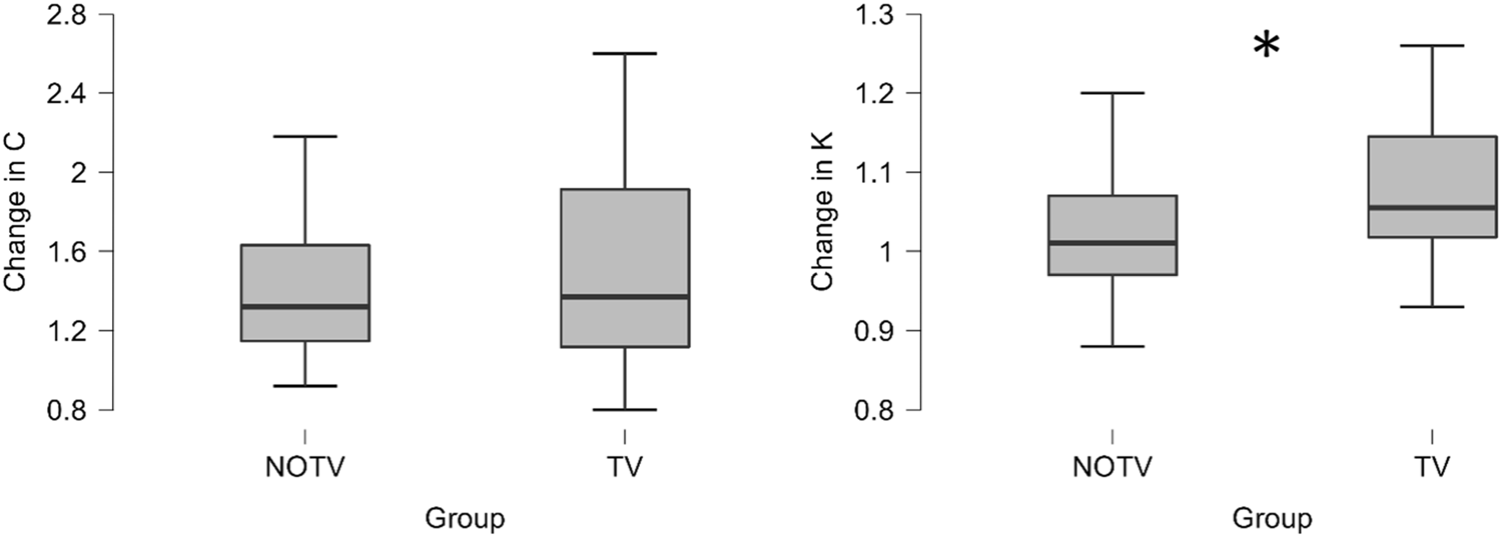
Change of visual processing speed C and VSTM capacity K in TVA. Note: Left: C_rc_ indicating the relative change from initial to final assessment in visual processing speed C. There was no significant difference in visual processing speed C between the groups, p > 0.05. Right: K_rc_ indicating the relative change from initial to final assessment in visual short-term memory capacity K. There was a significant difference in the change of visual short-term memory capacity K between initial and final assessment between groups, *p* = 0.005, *d* = − 0.757, 95%-CI[− 1.279, − 0.228]. Asterisk symbol (*) markes statistically significant differences between the groups.

For the whole report parameters, we found a statistically significant increase in visual processing speed *C* in both groups. Mean increase in NOTV was 14.35 letters per second from initial to final assessment, *z* = 10.00, *p* < 0.001. In TV we found an increase of 14.89, *z* = 48.00, *p* < 0.001. Relative change of C (*C*_*rc*_) and absolute change of C (*C*_*change*_) however did not differ statistically different between the groups, *U* = 539.00, *p* = 0.891, see Fig. 5.

**Figure 6:**
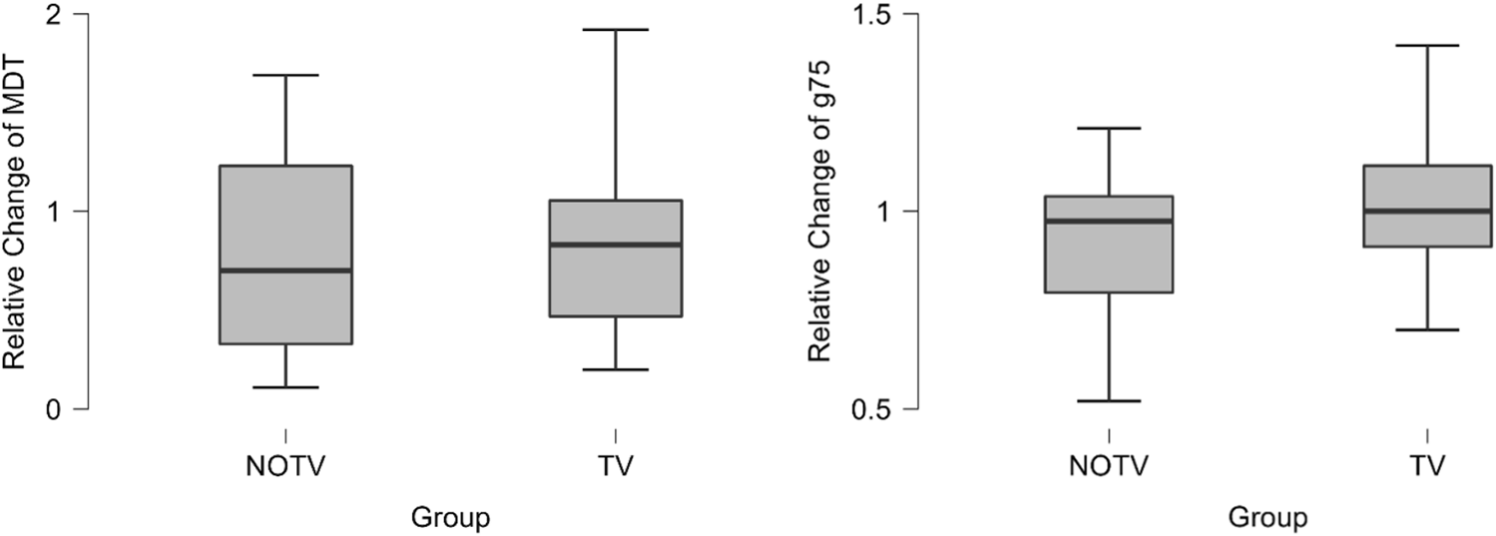
Somatosensory perception testing via relative change in MDT and GOT. Note: There was no significant difference between the groups in relative change of MDT in mN, *p* > 0.05. There was no significant difference between the groups in relative change of g75, representing GOT results, *p* > 0.05. Asterisk symbol (*) markes statistically significant differences between the groups.

While visual short term memory capacity *K* increased in both groups as well, the change in NOTV was low and not statistically significant (mean: + 0.048 maximum number of letters, *t*(32) = -0.759, *p* = 0.453) while it was high as well as statistically significant in TV (mean: + 0.174, *t*(31) = − 2.54, *p* = 0.016, *d* = − 0.448). Absolute change in visual short term memory capacity *K*_*change*_ was statistically significant higher in TV (mean: + 0.254, *t*(58) = − 3.19, *p* = 0.002, *d* = − 0.825, see Fig. 6). Relative change in visual short term memory capacity *K*_*rc*_ was statistically significant higher in TV (*t*(58) = − 2.92, *p* = 0.005, *d* = − 0.757, 95%-CI[− 1.279, − 0.228]).

The analysis of the partial report parameters revealed no statistically significant change or difference in top-down control *alpha* or spatial laterality *w_lat, p* > 0.05.

### Somatosensory perception

MDT data showed no significant difference between NOTV and TV as well as no difference between pre- and post-trial data within both groups (see Fig. 6 and Table 8 in the supplementary material). Relative difference of MDT did not differ between the groups, *U* = 714.50, *p* = 0.799.

GOT data revealed similar results of no significant difference between the groups and no difference between pre- and post-trial data within both groups (see Table 9 in the supplementary material). Relative difference of g75 did not differ between the groups, *t*(67) = − 3.11, *p* = 0.330, *d* = − 0.237.

## Discussion

Motor learning performance in our study—i.e., the acquisition of the motor skills necessary for touch-typing—was distinctly higher in TV compared to NOTV (see Fig. 3). Furthermore, the VSTM capacity was increased in TV compared to NOTV (see Fig. 5). RSN FC between the groups showed relevant changes as well. FC between VAN1 and DMN1 changed anticorrelated: in TV the FC increased distinctly while it decreased in NOTV. FC of VIS-A in TV (primarily the left extrastriate area) expanded its exchange with most areas of DMN1 (see Fig. 4). Task-related FC revealed a pre/post change in DAN1 and DAN2: FC decreased in TV while it increased in NOTV. The higher motor learning performance (characterized by MLE) in TV was accompanied by a higher intrinsic FC in left MOT-B. Grey matter volume in TV increased in the left entorhinal cortex while it decreased in the left angular and the right precentral gyrus. These results show changes which are most likely connected with our experimental intervention.

Until now, watching television is not yet known to influence adult motor learning performance or VSTM capacity. However, it is recognized that it can alter functional brain network connectivity and brain volume on principle^29,30^ even if applied for a short duration like in our intervention (i.e., 40 h in 5 days). The effect correlates positively with the duration of the stimulus (i.e., the amount of television watching). The mostly small effect sizes (~ 0.2) in FC changes in our study match this finding. Learning a new motor skill can change functional brain network connectivity as well, while the extent varies^31,32^. Additionally, learning how to touch-type imposes constant VSTM demands, as visually presented letters need to be processed, kept in VSTM, and typed. Thus, a reciprocal influence between VSTM and touch-typing training can be assumed.

We therefore suggest that the intervention (i.e., intense visual stimulation through television) induced a specific state of cerebral information exchange, in which resources were focused on the imminent motor learning task, which required to transform visual input (i.e., the letters) into associated motor output (i.e., typing the correct characters on the keyboard). We assume that this state induced improved visuomotor integration and caused superior performance compared to the control group. We consider it possible that the intervention led to an earlier completion of the skill acquisition stage of motor learning and therefore to a faster entry into the consolidation stage accompanied by a better learning performance.

The results of our experiment support those conclusions: RSN FC between VAN1 and DMN1 represents exchange between the precentral area and the mPFC (medial prefrontal cortex). Fingers are represented in this precentral area (Brodmann area 4p)^34^. Based on early lesion studies as well as modern methods of motor learning investigation combined with classical conditioning mPFC is thought to be involved in the integration of motor learning processes^35^. Their elevated FC in TV includes a more efficient and higher level of information exchange^36^. Left VIS-A of TV shows an increase in FC predominantly to different regions of the DMN1 compared to control. VIS-A consists of the secondary visual cortex in the extrastriate area and is known to respond to motor action following visual stimuli^37^ The DMN is accepted to be involved in goal-independent intrinsic activity^38^. The posterior precuneus cingulate cortex, an important element of the DMN, could be central for dividing internally (e.g., memory consolidation and recollection) and externally (e.g., changing the focus of attention) directed cognitive activity^39,40^. While there were effects on the right VIS-A, the focus of the detected changes in RSN FC on the left hemisphere could be explained by the known dynamic hemispheric asymmetry for visual information processing^41^, which however would rather let expect a focus in the right hemisphere^42^. The also observed increased VSTM capacity in TV however might suggest a dynamic adaptation to the visual information workload (i.e., television) which could have induced changes in the usually not-dominant left hemisphere^43,44^. VSTM workload however correlates predominantly with activity in IPS, which is not part of VIS-A but DAN^45,46^. Yet the visual trigger for motor action in the touch-typing learning sessions (i.e., the letters) were presented in the central visual field shared by both hemispheres, so the dominance of the right hemisphere should emerge as it is known to be able to cover the whole visual field^47–49^. The left hemisphere however is focused on the contralateral right visual field^41,50^. As the FC in RSN was measured before and after the intervention and not during the intervention, the detected changes are most likely to represent an adaptation to the induced demand. Further studies should be conducted analysing the FC changes induced by television viewing.

Additionally, grey matter volume pre/post comparison through VBM revealed a volume increase in the left entorhinal cortex in TV. This area is known for its role in episodic memory consolidation^51^ but is discussed for influencing (motor) learning processes as well^52^. De Brouwer et al.^52^ showed that higher performance in motor learning can be associated with higher volume in the entorhinal cortex. These findings match our results. However, the motor learning tasks were not similar in the two studies as participants in de Brouwer et al. trial were drawing a path with a stylus on a tablet. The entorhinal cortex volume increase might nevertheless facilitate memorizing the necessary movements for motor learning of touch-typing. The small effect sizes in RSN FC and VBM might be due to the relatively small sample size per group for non-behavioral testing.

Task-related FC change revealed a lowering of exchange between right DAN1 (right superior parietal lobule/IPS) and right DAN2 (temporal occipital pole) in TV while it increased in NOTV. Our other results would let expect inverted findings regarding right DAN1 and right DAN2. The superior parietal lobule is part of the secondary sensorimotor cortex. The IPS region is involved in the learning of finger motor sequence learning and visual attention^53,54^. Activity in the IPS for example is known to be of key relevance for the capacity of VSTM and correlates with its storing amounts^45,46^. Furthermore, activation of the IPS region correlates with the demand for the two aspects of finger motor sequence learning: movement execution and timing^53^. The right-sided focus of the detected differences in this area might be due to a hemispheric asymmetry with the right side involved in both movement execution and timing while the left side is primarily involved in movement execution^53^. A possible explanation for our findings is that hypoconnectivity inside the DAN has been linked to Attention-Deficit/Hyperactivity Disorder (ADHD)^55^, which leads to diminished capabilities of focus direction and stimulus sorting. Nevertheless, these changes were connected to a higher performance in touch-typing learning in TV in our trial. We suggest that the change in RSN FC is an effect of the intense long-term visual stimulation while the change in task-related FC is due to the task at hand (i.e., MST). A theoretical explanation approach is that a shift in visual information processing to a less filtered pathway might induce the DAN hypoconnectivity^56^.

Additionally, somatosensory perception investigated by MDT and GOT remained unaffected in our trial in both groups in comparison and within. The top-down processing of somatosensory information input (measured in GOT) seems not to be altered by visual stimulation through television and/or touch-typing training. MDT and its simple bottom-up mechanism is presumably too fast^57^ in adapting to be assessed with temporal distance to the visual stimulation. As the measurement of somatosensory perception was included in this study on the basis of the “Model of metamodal cortex”^23^, we expected reduced perceptional capabilities in the interventional group TV: redistribution of cognitive resources to satisfy the demands in information processing induced by visual stimulation through television could lead to lower performance in somatosensory perception^58^. In the light of the unaffected performance and supported by our FC RSN results we suggest that changes induced by our intervention and touch-typing learning were focused on other regions/networks of cortical activity like the extrastriate visual area. Areas relevant for somatosensory perception like primary somatosensory cortex were not affected in our fMRI studies. We suggest that our intervention did not reach the necessary extent of everyday alternation to impair fundamental sensory information processing like somatosensory perception.

We suggest that the intervention group TV entered a specific state of cerebral information processing because of intense visual stimulation and the continuous activation of visual information processing areas supported the interaction between handling of visual information and execution of the corresponding motor actions thus enabling better motor learning performance. A relevant aspect however is the fact, that the experimental procedure of our trial included not only an increased amount of visual stimulation through television for TV (from 180 min per day on average to 480 min per day, + 62.5%) but also a non-avoidable reduced amount of visual stimulation through television for NOTV (− 100%): Participants in NOTV usually and on average included 167 min per day of watching television in their daily routine. While planning the procedure of the study, the elimination of television for NOTV was the only feasible control group setting available. A group watching accustomed amounts of television was not practicable as control due to spatial and organisational constraints. This could be an influencing variable since it might function as an interventional alteration of daily routine. Anticorrelated changes in our FC results like the connectivity between right VAN-1 and right DMN-1 support this assumption. However, the main FC results which involve the change of left VIS-A interconnectivity is not anticorrelated. Additionally, the induced alteration of daily routine through increasing television viewing time by ~ 62.5% can be viewed as a much more impactful experimental intervention because it is rarely encountered outside of the experiment: Spending no time watching television is much more common in the participants age group than spending 480 min watching television. The average television viewing time for adults between 20 and 30 years of age is 75 min per day^3^. Our participants in NOTV tend to estimate themselves in the upper third of the distribution (mean: 167 min per day, see Table 2 in the supplementary material)), but so do the participants in TV (mean: 180 min per day, see Table 2 in the supplementary material). Nevertheless, NOTV might have encountered a deprivation of visual stimulation during our experiment, so a concluded assignment of the effects is not possible without further investigation. Additional groups with individualized daily routines including usual television viewing times might offer further insight. Groups and interventions depending on the participants usual television viewing times might also be an effective method to control for this variable.

Concerning other limitations of our study, our trial did not enable us to identify explicit causal interactions between motor learning performance and visual stimulation due to the necessary temporal segregation of these aspects. Additional groups with different intensities of visual stimulation or application of visual stimulation while performing fMRI would enable subsequent experiments to address these limitations. Cranial magnetic resonance spectroscopy in combination with visual stimulation might also provide insight into relevant causal neural mechanisms. Furthermore, visual stimulation based on television does additionally contain auditory information. This might influence FC in the analysed networks. Subsequent experiments could be designed to rely on visual media accompanied by minimal auditory information. Including participants of other age groups (e.g., seniors or teenagers) might also help to contextualise our findings.

## Methods

### Experimental design

**Figure 7:**
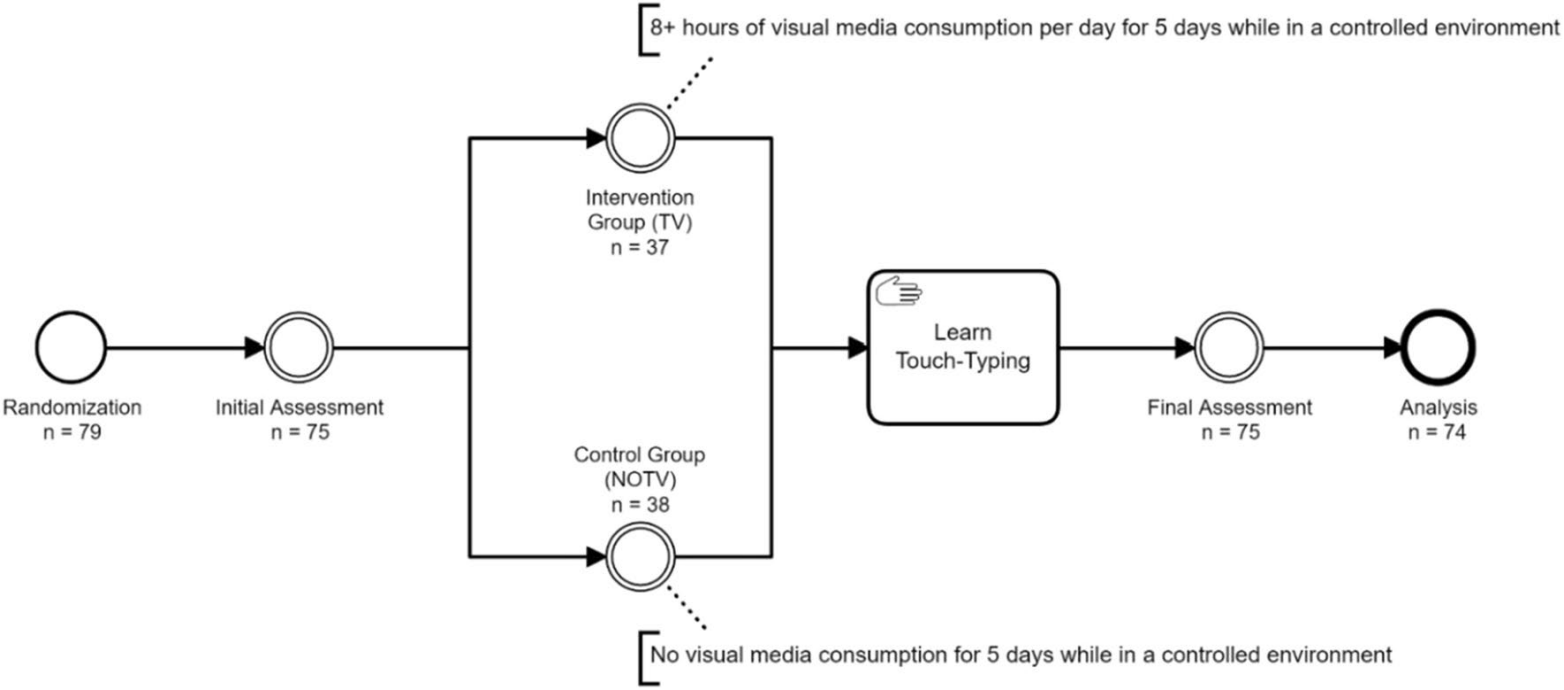
Study design. Note: Trial design and procedure (TV Studie Jena).

The study was designed as a randomized controlled intervention trial. Based on power analysis applying the data of our pilot trial (n = 8) the estimated minimal necessary sample size was a total of 68 subjects. Seventy-nine subjects were recruited. 4 participants were excluded before initial assessment because of illness and 1 subject after the final assessment due to missing data. Subjects were randomized in one of two groups: the experimental group (TV, n = 39) watched a minimum of 8 h (mean = 8.09 h/day, range = 8.00–8.75 h/day) of visual media like television per day. The control group (NOTV, n = 40) did not consume any visual media. Both groups were accommodated in a controlled environment for 5 days and meanwhile completed a course in touch-typing on the PC keyboard. Touch-typing performance served as motor learning surrogate, as this ability was new to all participants. Before and after the experiment we conducted assessments (pre- and post-assessment) on resting-state functional connectivity (fMRI), visual attention (TVA), somatosensory perception (MDT and GOT), see Fig. 7 below and Table 1 in the supplementary material for further information regarding trial procedure.

### Participants

We recruited 79 subjects between 20 and 30 years of age (*M* = 22.9 years, *SD* = 2.49, *n* female = 40). Calculated sample size based on power analyses was 68. All subjects were Eurasian students at the Friedrich Schiller University Jena or college of higher education in Jena, Germany. Inclusion criteria were high school graduation (*Abitur*, i.e., aptitude for attendance to university) and age between 20 and 30 years. Exclusion criteria were neurological or psychiatric disorders and experience/skill in touch-typing (10-finger-system) on a QWERTZ PC-keyboard. Furthermore, we did not include professional musicians or athletes due to possibly altered motor learning capabilities.

All subjects were informed about the procedure of the trial in written form as well as personally and gave their written consent according to the Declaration of Helsinki II. The trial was approved by the ethics committee of the medical faculty of the Friedrich-Schiller-University Jena, Germany (Registration number: 2018-1060-BO). All methods were carried out in accordance with relevant guidelines and regulations.

Media consumption habits and demographical data were collected at the initial assessment. In TV the daily television consumption was increased by 62.5% through our intervention (from 180 min per day on average to 480 min per day). In NOTV the daily television consumption was decreased by 100% (from 167 min per day on average to 0 min per day). Subjects were screened for symptoms of depression by *Beck’s depression inventory* (BDI-II)^59^ and excluded if scoring above 8 points, no subjects were excluded hereby. All participants had normal or corrected-to-normal vision and were not colour-blind. Six recruited subjects were left-handed, all other participants were right-handed as ascertained by the *Edinburgh Handedness Inventory*^60^. The participants were paid for taking part in the study.

All subjects were randomized either into the intervention (TV, age: *M* = 22.32 years, *SD* = 2.27) or into the control group (NOTV, age: *M* = 23.46 years, *SD* = 2.60). Randomization was achieved using simple randomization by computer generated random numbers to assign participants to intervention or control group. For details regarding the participants see Table 2 in the supplementary material. Four subjects were ruled out after the randomization because of illness (two of each group). One subject (NOTV) was ruled out after final assessment because of missing data (NOTV).

### Procedure and controlled environment

The intervention of this trial took place over five days during which the subjects were accommodated in a controlled environment. This was necessary to prevent distraction and reproduce the same environment for all subjects. Both the control and the intervention group stayed in a hostel in Jena (Germany) for 5 days and 4 nights. We established a set of behavioural rules for the controlled environment. Adherence to those rules was checked regularly, randomly, and unannounced multiple times per day throughout the trial by members of the study team. There were no major violations of these rules. We tested the viability of our procedure with eight subjects in a pilot trial and optimized it afterwards. The data of these subjects are not included in the analysis. All subjects were allowed to spend 90 min each day outside the hostel. Any physical activity (like Walking, Jogging, Yoga, etc.) had to be performed in this time window. The performed physical activity was not allowed to exceed the usual extent of the individual. The physical activity was recorded and monitored. For the rest of the time, they had to stay either in their rooms, the common room, or in the lounge areas of the hostel. Smartphone or cell phone use was allowed for only 30 min a day, however talking via phone was possible without restriction. The following activities were not allowed for any of the groups: playing video games, learning new motor skills like juggling, or knitting, consuming drugs of any kind (except cigarettes) and using touch-typing outside of training times while handwriting was allowed. All subjects had to record their activities and times of absence. All participants shared the same meals and mealtimes. The intervention group had to consume eight hours of visual media content (i.e., television or video-on-demand-services) of their choice accumulatively per day in a passive posture (i.e., sitting or lying). The intervention group had to keep record of their watched media content (e.g., genres of watched content, duration). The control group was allowed to perform all usual leisure-time activities, which were not new to them neither included screen time. Most participants choose reading, board games, and cooking.

Every day at the same time in the morning and in the afternoon, there was a 45-min grouped and supervised training session in touch-typing for the participants (see next section for more details), resulting in 90 min of training per day. The day before and the day after leaving the controlled environment, each subject underwent assessment (pre and post) for data acquisition. For the exact pattern of our trial procedure please refer to Table 1 in the supplementary material.

### Touch-typing

While accommodated in the controlled environment, all subjects underwent a five-day long course on typing on a keyboard with all ten fingers (touch-typing, 10-finger-system-typing) on the keyboard layout QWERTZ. In the 10-finger-system of touch-typing each character on the keyboard has a defined finger assigned to it (e.g., F: index finger left), whereas self-taught typing-styles vary heavily. All subjects completed their course with the free Tipp10® software (Thielicke IT Solutions, Version 2.1) on the same type of notebook with the same type of keyboard (Lenovo® Thinkpad T60). The lessons were identical in content and therefore in skill demands for both groups. Two 45-min sessions of training were applied each day. The difficulty of typing regarding motor skill requirements was increasing over the five days of the trial through expanding letter variance and word complexity. The course was always supervised by trained personal to guarantee a correct procedure, including the use of all ten fingers and correct finger-character combinations. No feedback was given to subjects concerning their typing performance. Consequential errors were avoided by the used software as only the correct letter input would enable progress. Quiet surroundings to avoid distraction or disturbance were established by members of the study team.

Each day, the first session took place from 9 to 9.45 or 9.45 to 10.30 AM with the two groups alternating between the dates. The second session took place from 3 to 3.45 or 3.45 to 4.30 PM with the two groups alternating between the dates as well. The two groups trained in direct succession and separately. Every training session consisted of a repeated sequence of a total of 18 different lessons following a set schedule. Three different and consecutive lessons of 3 min each were followed by a break of 2 min. This sequence was repeated three times before the final sequence concluded the session without a following break. Thus, each session consisted of 12 lessons and 3 breaks, resulting in an overall 42-min duration. A 3-min buffer was included for possibly necessary explanations from the supervising personnel, summing up to 45 min total duration for one session. The difficulty was steadily increased over the subsequent lessons by introducing new characters (except the last lesson, which included all characters), areas on the keyboard and by increasing the distance between characters. The frequency of each lesson throughout the course was determined in advance by its contents’ usefulness to promote motivation and progress. The content, procedure, and duration of first (morning) and second (afternoon) daily session was identical. Thus, the participants were trained through 24 3-min-long lessons per day separated into 2 daily sessions.

Lesson 3 (L3) was selected beforehand as a marker for the training effect and was carried out repeatedly and in each training session over the trial. On the first day, this marker lesson was completed 4 times in the morning training session and 4 times in the afternoon training session, as it was part of the normal training in addition to being a training effect marker. On each of the following four days, the marker lesson was completed twice per day—once in the morning training session and once in the afternoon training session. This specific lesson was chosen, because it occurred early in the course and both pilot trial subjects and study team judged it to be neither hard nor easy. The subjects were unaware of the specific function of this lesson.

The baseline typing skill (i.e., general agility of hand and fingers) were (a) the number of correctly typed characters in 1 min (correct characters per minute, *ccpm*) in the dictation at initial assessment and (b) *ccpm* in the first training session (day 1).

### Touch-typing performance parameters

Touch-typing performance was measured by three values, namely: Gain_L3_, Daily Improvement (DI) and Motor Learning Efficiency (MLE). These scores were determined after the pilot trial to be the most reasonable benchmarking values to measure motor skill learning performance. The different days of the study (1–5) served as within-subject factors while the group served as between-subject factor.

The gain in motor skill regarding touch-typing (${{\text{Gain}}}_{{\text{L}}3}$) was measured by the increase in correctly entered characters during the marker lesson (L3) in relation to the first day of the trial. ${{\text{Gain}}}_{{\text{L}}3}$ for the comparison of day 1 to day 5 would therefore be called ${{\text{Gain}}}_{{\text{L}}{3}_{{\text{D}}5{\text{toD}}1}}$. Performance in L3 was computed as mean correctly entered characters per day. This value was divided by the value of the first day, resulting in a measurement for the change in correct entered characters between the two days. ${{\text{Gain}}}_{{\text{L}}{3}_{{\text{D}}1{\text{toD}}1}}$ always resulted in 1. Please see formula below.

Daily Improvement was calculated by the division of overall correctly entered characters in the second daily session by the overall correctly entered characters in the first daily (i.e., morning) session. Please see formula below.

Motor Learning Efficiency (MLE) was calculated by summing up Gain_L3_ and DI before dividing the calculated value by the number of included values (10, Gain_L3_ and DI for 5 days each). MLE combines within-day (DI) and between-day (Gain_L3_) data for a general numeric representation of motor learning performance. Please see formula below.

Formula Gain_L3_:

$${\text{Gain}}_{{{\text{L}}3_{{{\text{DXtoD}}1}} }} = \frac{{mean\left( {entered\; input - errors} \right) \;day X}}{{mean\left( {entered \;input - errors} \right)\; day 1}}$$

Example GainL3:

$${\text{Gain}}_{{{\text{L}}3_{{{\text{D}}3{\text{toD}}1}} }} = \frac{{mean\left( {\left( {200 - 10} \right) + \left( {210 - 12} \right)} \right) day 3}}{{mean\left( {\left( {100 - 15} \right) + \cdots + \left( {110 - 14} \right)} \right) day 1}} = \frac{185}{{100}} = 1.85$$

Representing an increase of 85% in correct entered characters in L3 between day 1 and day 3.

Formula DI:

$${\text{DI}} = \frac{correctly\; entered \;characters \;session\, 2 \;day \,X}{{correctly\; entered\; characters\; session \,1 \;day\, X}}$$

Formula MLE:

$$MLE = \frac{GainL3\,\, Day 1 + GainL3 \,\,Day 2 + \cdots DI\,\,Day 1 + DI \,\,Day 2 + \cdots }{{10}}$$

### Touch-typing—subgroups

To look for potential confounding factors, we conducted analysis concerning the influence of age, gender, pre-trial media consumption habits and media content in the study. We calculated Pearsons’s correlation coefficient to investigate the influence of age on performance in touch-typing represented by Gain_L3_, DI and MLE. To look for potential influence of pre-trial media consumption on MLE, we conducted a one-way ANOVA. All subjects were split up in three groups by their pre-trial media consumption: Low (0–15 h/week, *M* = 9.74, *SD* = 3.36), Average (15–30 h/week, *M* = 21.56, *SD* = 3.37) and High (30+ h/week, *M* = 36.60, *SD* = 5.64). To evaluate the potential influence of media content on MLE, we conducted a one-way ANOVA. We separated the subjects of TV for their choice of media content which they consumed during the study. We created three groups: Group 1 (Relax) contained subjects who watched mostly relaxing or educative content (Education, Documentation, Sitcom, Reality-TV, Sports, Music, Fantasy, Children) while Group 3 (Thrill) consisted of those who had chosen mostly thrilling or exciting media (Drama, Action, Thriller, Crime, Animation). Subjects who watched both types equally were assigned to Group 2 (Mixed).

### fMRI data acquisition

fMRI delivers functional data (i.e., for example functional connectivity) of the brain’s activity. Functional connectivity is defined as statistical dependency between spatially separated cerebral events. It is based on temporal correlation of low frequency fluctuations of BOLD-signal between spatially separated brain regions in task-related or resting-state fMRI^61,62^. It serves as a surrogate for information-exchange between these areas and their functions^63^. Functional MR-Imaging uses the BOLD-contrast to visualise task-induced activity of brain areas (e.g., Manual Sequence Task, MST) or neuronal networks in resting-state (RSN).

MRI data were acquired twice—before and after the experimental phase—of each participant using a 3T MR scanner (AG Prisma 3, Siemens, Erlangen, Germany) and a standard quadrature 64-channel phased array head coil.

We obtained high-resolution T1-weighted structural images by axial 3-dimensional Magnetization Prepared RApid Gradient Echo sequence (MP-RAGE, for the parameters of the MP-RAGE sequence see Table 3 in the supplementary material), functional MR imaging data during rest (resting-state fMRI, rs fMRI) and during a task (i.e., task-related brain activity, stim fMRI). For the functional MRI data, we used a gradient-echo echo-planar imaging sequence sensitive to blood oxygenation level dependent contrast (GE EPI-BOLD). For the parameters of the EPI-BOLD sequence see Table 3 in the supplementary material. We used Simultaneous Multi Slice (SMS) technique for Multiband image acquisition.

The order of MRI sequences during data acquisition was: (1) Scout, (2) rs fMRI, (3) stim fMRI, (4) MP-RAGE T1.

During resting-state data acquisition, the subjects were instructed to lie still, close their eyes and to stay awake. During the task-related data acquisition the participants performed a Motor Sequence Task (MST)—also called Finger Tapping Task (FTT): Whilst in the MRI and during the task-related data-acquisition the participants were presented with a constant sequence of numbers through a mirror-monitor-combination (4, 1, 3, 2, 4). This numbers represented 4 fingers on the left hand which rested on an input pad with 4 buttons. The thumb was not included. Pressing these buttons as fast and correct as possible was the goal of this experiment. The active number was indicated. The active number only moved forward if correctly entered. The task lasted 12 min, consisting of 12 blocks each containing 30 s of activity and 30 s of rest. Numbers were only presented during activity.

### fMRI analysis cortical parcellation

The cortical parcellation atlas by Yeo et al.^28^ was used to define 17 functional brain networks which represent 17 resting-state networks (RSN) created by seed-based correlation analysis. These networks (17N Yeo, see Table 4 in the supplementary material) are a sub-parcellation of 7 previously identified functional networks (7N Yeo^28^). RSN were shown to be consistent^64^ while taxonomy differs. Functional connectivity (FC) was calculated as the correlation between these functional networks as well as within these networks. We chose the following functional networks for our hypotheses because of their relevance for motor learning^62,65^ and processing of visual information^33^: Occipital networks Visual 1 and 2 (VIS1, VIS2), pericentral networks Motor 1, 2 and 3 (MOT1, MOT2, MOT3), midcingulo-insular Ventral Attention Network 1 and 2 (VAN1, VAN2), medial frontoparietal Default Mode Network 1, 2 and 3 (DMN1, DMN2, DMN3), dorsal frontoparietal Dorsal Attention Network 1 and 2 (DAN1/DAN2), and lateral Frontoparietal Network 1, 2, 3 and 4 (FP1, FP2, FP3, FP4).

### fMRI data analysis

The acquired fMRI resting-state data was analysed on a Windows® 10 (Microsoft, USA) PC using MATLAB (MathWorks®, Natick, MA, Version R2020a). Results included in this manuscript come from analyses performed using CONN^66^ (RRID:SCR_009550) release 22a^67^ and SPM^68^ (RRID:SCR_007037) release 12.7771. The following methods reports was extracted from CONN 22a.

#### Resting-state fMRI data analysis

##### Preprocessing (CONN methods report)

 Functional and anatomical data were pre-processed using a flexible preprocessing pipeline^69^ including realignment with correction of susceptibility distortion interactions, slice timing correction, outlier detection, direct segmentation (grey matter, white matter, cerebrospinal fluid) and MNI-space normalization, and smoothing. Functional data were realigned using SPM realign & unwarp procedure^70^, where all scans were coregistered to a reference image (first scan of the first session) using a least squares approach and a 6 parameter (rigid body) transformation^71^, and resampled using b-spline interpolation to correct for motion and magnetic susceptibility interactions. Temporal misalignment between different slices of the functional data was corrected following SPM slice-timing correction (STC) procedure^72,73^, using sinc temporal interpolation to resample each slice BOLD timeseries to a common mid-acquisition time. Potential outlier scans were identified using ART^74^ as acquisitions with framewise displacement above 0.9 mm or global BOLD signal changes above 5 standard deviations^75,76^, and a reference BOLD image was computed for each subject by averaging all scans excluding outliers. Functional and anatomical data were normalized into standard MNI space, segmented into grey matter, white matter, and CSF tissue classes, and resampled to 1.4 mm isotropic voxels following a direct normalization procedure^76,77^ using SPM unified segmentation and normalization algorithm^69,78^ with the default IXI-549 tissue probability map template. Last, functional data were smoothed using spatial convolution with a Gaussian kernel of 6 mm full width half maximum (FWHM).

##### Denoising (CONN methods report)

 In addition, functional data were denoised using a standard denoising pipeline^69^ including the regression of potential confounding effects characterized by white matter timeseries (5 CompCor noise components), CSF timeseries (5 CompCor noise components), motion parameters and their first order derivatives (12 factors)^79^, outlier scans (below 59 factors)^75^, session and task effects and their first order derivatives (8 factors), and linear trends (2 factors) within each functional run, followed by bandpass frequency filtering of the BOLD timeseries^80^ between 0.008 and 0.09 Hz. CompCor^81,82^ noise components within white matter and CSF were estimated by computing the average BOLD signal as well as the largest principal components orthogonal to the BOLD average, motion parameters, and outlier scans within each subject's eroded segmentation masks. From the number of noise terms included in this denoising strategy, the effective degrees of freedom of the BOLD signal after denoising were estimated to range from 234.7 to 278.9 (average 271.2) across all subjects^76^.

##### First-level analysis (CONN methods report)

 RRC_01: ROI-to-ROI connectivity (RRC) matrices were estimated characterizing the functional connectivity between each pair of regions among 121 ROIs. Functional connectivity strength was represented by Fisher-transformed bivariate correlation coefficients from a general linear model (weighted-GLM^69^), estimated separately for each pair of ROIs, characterizing the association between their BOLD signal timeseries. Individual scans were weighted by a boxcar signal characterizing each individual task or experimental condition convolved with an SPM canonical hemodynamic response function and rectified.

##### Group-level analyses (CONN methods report)

Were performed using a General Linear Model (GLM^69^). For each individual connection a separate GLM was estimated, with first-level connectivity measures at this connection as dependent variables (one independent sample per subject and one measurement per task or experimental condition, if applicable), and groups or other subject-level identifiers as independent variables. Connection-level hypotheses were evaluated using multivariate parametric statistics with random-effects across subjects and sample covariance estimation across multiple measurements. Inferences were performed at the level of individual clusters (groups of similar connections). Cluster-level inferences were based on parametric statistics within- and between- each pair of networks (Functional Network Connectivity^83^), with networks identified using a complete-linkage hierarchical clustering procedure^84^ based on ROI-to-ROI anatomical proximity and functional similarity metrics^69^. Results were thresholded using a combination of a p < 0.05 connection-level threshold and a familywise corrected p-FDR < 0.05 cluster-level threshold^85^. Effect size is displayed as Fisher-transformed groupwise difference in connectivity *beta*.

#### Task-related fMRI data analysis

##### Preprocessing (CONN methods report)

 Functional and anatomical data were pre-processed using a flexible preprocessing pipeline^69^ including realignment with correction of susceptibility distortion interactions, outlier detection, direct segmentation and MNI-space normalization, and smoothing. Functional data were realigned using SPM realign & unwarp procedure^70^, where all scans were coregistered to a reference image (first scan of the first session) using a least squares approach and a 6 parameter (rigid body) transformation^71^, and resampled using b-spline interpolation to correct for motion and magnetic susceptibility interactions. Potential outlier scans were identified using ART^74^ as acquisitions with framewise displacement above 0.9 mm or global BOLD signal changes above 5 standard deviations^75,76^, and a reference BOLD image was computed for each subject by averaging all scans excluding outliers. Functional and anatomical data were normalized into standard MNI space, segmented into grey matter, white matter, and CSF tissue classes, and resampled to 2 mm isotropic voxels following a direct normalization procedure^76,77^ using SPM unified segmentation and normalization algorithm^78,86^ with the default IXI-549 tissue probability map template. Last, functional data were smoothed using spatial convolution with a Gaussian kernel of 8 mm full width half maximum (FWHM).

##### Denoising (CONN methods report)

 In addition, functional data were denoised using a standard denoising pipeline^69^ including the regression of potential confounding effects characterized by white matter timeseries (5 CompCor noise components), CSF timeseries (5 CompCor noise components), motion parameters and their first order derivatives (12 factors)^79^, outlier scans (below 223 factors)^75^, session and task effects and their first order derivatives (4 factors), and linear trends (2 factors) within each functional run, followed by bandpass frequency filtering of the BOLD timeseries^80^ between 0.008 and 0.09 Hz. CompCor^81,82^ noise components within white matter and CSF were estimated by computing the average BOLD signal as well as the largest principal components orthogonal to the BOLD average, motion parameters, and outlier scans within each subject's eroded segmentation masks. From the number of noise terms included in this denoising strategy, the effective degrees of freedom of the BOLD signal after denoising were estimated to range from 207.3 to 233.7 (average 229.9) across all subjects^76^.

##### First-level analysis (CONN methods report)

 SBC_01: ROI-to-ROI connectivity (RRC) matrices were estimated characterizing the functional connectivity between each pair of regions among 121 ROIs. Functional connectivity strength was represented by Fisher-transformed bivariate correlation coefficients from a general linear model (weighted-GLM^69^), estimated separately for each pair of ROIs, characterizing the association between their BOLD signal timeseries. Individual scans were weighted by a boxcar signal characterizing each individual task or experimental condition convolved with an SPM canonical hemodynamic response function and rectified.

##### Group-level analyses (CONN methods report)

 Were performed using a General Linear Model (GLM^69^). For each individual connection a separate GLM was estimated, with first-level connectivity measures at this connection as dependent variables (one independent sample per subject and one measurement per task or experimental condition, if applicable), and groups or other subject-level identifiers as independent variables. Connection-level hypotheses were evaluated using multivariate parametric statistics with random-effects across subjects and sample covariance estimation across multiple measurements. Inferences were performed at the level of individual clusters (groups of similar connections). Cluster-level inferences were based on parametric statistics within- and between- each pair of networks (Functional Network Connectivity^83^), with networks identified using a complete-linkage hierarchical clustering procedure^84^ based on ROI-to-ROI anatomical proximity and functional similarity metrics^69^. Results were thresholded using a combination of a p < 0.05 connection-level threshold and a familywise corrected p-FDR < 0.05 cluster-level threshold^85^.

### Voxel-based Morphometry (VBM) analysis

For voxel-based morphometry (VBM) analysis^86^, we used the Computational Anatomy Toolbox 12 (CAT12, Structural Brain Mapping group, Jena University Hospital, Jena, Germany)^87^ for the MATLAB (MathWorks®, Natick, MA, Version R2020a) software package Statistical Parametric Mapping 12 (SPM12, Institute of Neurology, London, UK)^68^. We used the standard Neuromorphometrics anatomical atlas^88^. Longitudinal pre-processing protocol was applied to T1-weighted images of the participants from before and after the experiment. This included: automated quality insurance protocol, visual quality check, bias-field inhomogeneities correction, spatial normalisation, and segmentation into grey matter (GM), white matter (WM) and cerebrospinal fluid (CSF). Segmentation included accounting for partial volume effects, application of an adaptive maximum of a posterior estimations and utilisation of a hidden Markov Random Field model. For artefact exclusion on the border between grey and white matter we applied an internal grey matter threshold of 0.2.

Statistical group comparison was realised using the general linear model (GLM) in CAT12. We included age and gender as nuisance variables in each GLM to remove variance related to these variables. We first performed whole-brain analyses at a threshold of *p* < 0.001 (uncorr.). Afterwards we analysed areas included in the hypotheses as well as outside of these areas as an exploratory approach.

### Assessment of parameters of visual attention based on the theory of visual attention (TVA)

To compare visual information processing between the groups we used a computational modelling test method based on Bundesen’s Theory of Visual Attention (TVA)^26,89^. TVA postulates that visual information is processed in parallel. After an initial, unselective wave, visual objects are assumed to race towards visual short-term memory in a biased competition. The winner of the competition enter into visual short-term memory (VSTM) and are, thus, selected, consciously represented and available for further processes such as verbal report^90,91^. The VSTM serves as a short-term (i.e., several seconds) and low-capacity (i.e., ~ 4 objects in adults^92^) buffer for visual information^93^. The race of visual information can be quantified by algorithms, resulting in a set of parameters as individual estimates for each participant. The physiological basis for TVA was proposed 2005 by Bundesen as NTVA (Neural Theory of Visual Attention)^94^.

To assess parameters of visual attention we used TVA-based paradigms of whole and partial report. Penning et al.^95^ gave a summary of the basic principles of TVA^26^:

“In TVA-based psychophysical paradigms, participants are briefly presented with letter arrays and instructed to report either all or only specific letters (whole and partial report, respectively). By modelling report accuracy as a function of effective exposure time, researchers can estimate core visual attention parameters (i.e., VPS, visual short-term memory [VSTM] capacity, visual threshold, and top-down control) mathematically independently from each other. Empirical investigations^96,97^ support the assumption that the parameters obtained represent relatively dissociable processes. Because the report is verbal without speed stress, performance is determined by perceptual, rather than motor, capabilities.”

The subjects were briefly presented with a series of red and/or blue letters on a PC display. All (whole report) or red (partial report) letters had to be reported. Based on the accuracy of the letter report (dependent variable), different basic parameters determining visual attention performance of a given subject can be estimated.

Whole and partial report were carried out in direct succession at both assessments.

Subjects completed first the whole report and afterwards the partial report within 60 min. In the whole report the task was to report all presented red and blue letters. The letters were presented for variable exposure durations between 10 and 200 ms (independent variable). In the partial report subjects had to report only the recognized red letters while blue letters served as distractors. The exposure duration was individualized in a pre-test phase to correct for baseline differences (partial report only) aiming for a 70% success rate in identifying at least one letter correctly.

Letters were always arranged on a (non-visible) circle around a fixation cross on a 100 Hz-Flatscreen. The subject’s head was fixed in 60cm from the display by a skin rest. Letters were masked in half of the whole report trials and in all partial report trials by a red and a blue shape after presentation in order to delete the contents of the visual buffer^96^.

Based on maximum likelihood modelling of letter report accuracy at different exposure durations in the whole report, we calculated three parameters of visual processing: *t0*, *C* and *K*. The visual threshold *t0* represents the minimal exposure duration necessary for start of visual processing. Visual processing speed *C* is the individual processing rate given in visual objects per second. Visual short-term memory (VSTM) capacity *K* denotes the maximum number of objects that can be kept in VSTM at a given instant. The assessment of visual threshold *t0* served for validation of the whole report parameters *C* and *K*. *t0* is of no further interest concerning the aim of our study. Therefore, it was not further analysed after a baseline comparison across groups.

Based on the accuracy of letter report in the different partial report conditions, a calculation of spatial laterality *w_lat* and top-down control *alpha* is possible. While *w_lat* indicates whether participants allocate attentional weights to the left and right side of space in an equal manner, top-down control *alpha* indexes the ability to prioritize relevant (target) over irrelevant (distractor) information, with lower values indication better selectivity.

TVA parameters were tested for a statistically significant change between initial and final assessment (effect of Time) by paired samples t-test or Wilcoxon signed-rank test. Afterwards, the change of the parameters (*C*_*change*_ = *C*_*post*_−*C*_*pre*_ and *K*_*change*_ = *K*_*post*_−*K*_*pre*_, and relative change *C*_*rc*_ = *C*_*post*_/*C*_*pre*_ and *K*_*rc*_ = *K*_*post*_/*K*_*pre*_) was compared between the groups by independent samples t-test or Mann–Whitney-U test.

### Somatosensory perception

#### Grating-orientation-task (GOT)

To acquire data regarding the somatosensory perception of the subjects and a possible change, we carried out measurements via GOT using plastic gratings equivalent to J.V.P.-Domes®^98^ and Mechanical-Detection-Threshold (MDT) using Von-Frey-Hairs®. Both tests aim for determination of the somatosensory perception level of an individual via touching the skin of the dominant hand. GOT presents plastic grooves of various width (ranging from 3.5 to 0.5 mm) to the immobilized fingertip of the index finger of the dominant hand while the subject is blindfolded. The alignment of the grooves (transversal or longitudinal) is changed randomly, and each width is presented ten times. The subject reports back the felt alignment until performance drops below 75% (8/10) of correctly reported sensations. The principle is based on the J.V.P.-domes®^98^ but was adapted to a self-made construct for automatic stimulus delivery. For further analysis, the g75-value is calculated as a surrogate value for the groove spacing on which the subject would have scored with 75% accuracy, had it been presented^99^ (see formula below). 75% accuracy is considered an acceptable compromise between chance and perfect performance. GOT with J.V.P.-domes is considered to have very high sensitivity and specificity^100^ for sensory deficits. Two subjects had to be excluded from analysis because of mechanical malfunction of the device.

$${\mathbf{g}}_{{{\mathbf{75}}}} = {\text{ g}}_{{{\text{low}}}} + \, \left( {\left( {0.{75 } - {\text{ p}}_{{{\text{low}}}} } \right) \, \div \, \left( {{\text{p}}_{{{\text{high}}}} - {\text{ p}}_{{{\text{low}}}} } \right)} \right) \, \times \, \left( {{\text{g}}_{{{\text{high}}}} - {\text{ g}}_{{{\text{low}}}} } \right)$$

g = grating spacing, p = trials correct/n, n = number of trials (= 10), high = lowest grating spacing with 75% accuracy, low = highest grating spacing with 75% accuracy.

#### Mechanical detection threshold (MDT)

MDT was determined using a standardized set of Von-Frey-Hairs® (Optihair2, Marstock Nervtest, Germany)^101,102^. These plastic filaments apply pressure between 0.25 and 8 mN (grating-factor: 2) when they touch the skin of the dominant backhand on a hairless spot for 1 s while being bended to S-shape. All subjects were blindfolded and received the stimulation on the same area. Two threshold determinations (10 stimuli each) were acquired by alternately descending until the subject failed to notice the stimulus and ascending until re-noticing occurred (“method of limits”). Means and standard errors of the results of all blocks were calculated and analysed as surrogates for the actual threshold. Two subjects had to be excluded from analysis because of mechanical malfunction of the device.

### Dictation

To get used to the touch-typing learning software Tipp10®, to verify the claim of being unskilled in touch-typing as necessary for inclusion and to assess the general agility while typing on a PC keyboard as a baseline-measurement, the subjects had to complete a supervised 10-min dictation at the initial and final assessment. Content of this dictation was the fairy tale “Der Wolf und die Sieben Geißlein” (The wolf and the seven little goats) of the brothers Grimm. It was not chosen as validated test content but because it is a known and entertaining story, which enabled us to assess the touch-typing skills of the subject. The subjects were unaware of this affirmation of fulfilment of the inclusion criteria. The first dictation had to be completed in the individually accustomed way of typing on a PC keyboard. The second dictation had to be executed by touch-typing. Supervising personnel was present to assure compliance with those rules. Afterwards the number of correctly entered characters per minute (corr/m) was calculated and analysed.

### Statistical analysis

Statistical analyses were performed using IBM SPSS27® (Version: 27.0.0.0). Data was tested for normal distribution by Shapiro–Wilk test. Where normal distribution was attained, we used independent/paired samples t-test. Where normal distribution was not attained, the Mann–Whitney-U test was used. Data was tested for equal sphericity by Mauchly’s test. Where equal sphericity was not attained, Greenhouse–Geisser correction was applied. Data was tested for homogeneity of variance by Levene’s test. Touch typing data (Gain_L3_, DI and MLE) was analysed by independent samples t-test, Pearson’s correlation coefficient, one-way analysis of variance (ANOVA) as well as repeated-measures mixed ANOVA (mANOVA) with Greenhouse–Geisser correction and Tukey corrected post-hoc analysis. Groups served as between-subject factors (2 levels) and trial days as within-subject factors (5 levels). Data of the TVA-based assessment was evaluated by independent and paired samples t-test. The results of somatosensory perception (MDT & GOT) and dictation were assessed by independent samples t-test between groups and paired samples t-test within groups.

For the statistical analyses used in the fMRI and VBM data please see respective methods sections above.

Findings were considered significant at *p* < *0.05* (two-sided). Standard confidence interval (95% CI: mean ± z * SD/sqrt(n)) was used. Regarding fMRI analysis findings were considered significant at *p* < *0.05* (one-sided, FDR-corrected).

### Supplementary Information

Supplementary Information.

## Data Availability

The preregistration for our study can be accessed at https://www.drks.de/drks_web/setLocale_EN.do. Note that due to the pilot trial for procedure evaluation the registration is marked as retrospectively registered.
