# Supplementary material for: Visual stimulation by extensive visual media consumption can be beneficial for motor learning
Source: Sci Rep. 2023 Dec 12;13:22056. doi: 10.1038/s41598-023-49415-4 (PMC10716399; doi:10.1038/s41598-023-49415-4)
Supplement: Supplementary file 1 — Supplementary Information. [file 41598_2023_49415_MOESM1_ESM.pdf]

Supplementary material

Tables

Table 1

Trial Procedure

Group	Day 0	Day 2	Day 3	Day 4	Day 5	Day 6	Day 7
NOTV	Initial assessment:<br>1. fMRI<br>2. MDT<br>3. GOT<br>4. Dictation<br>5. TVA<br>6. questionnaires	CE<br>No media<br>90min Training	CE<br>No media<br>90min Training	CE<br>No media<br>90min Training	CE<br>No media<br>90min Training	CE<br>No media<br>90min Training	Final assessment:<br>Identical to initial assessment
TV		CE<br>8h+ media<br>90min Training	CE<br>8h+ media<br>90min Training	CE<br>8h+ media<br>90min Training	CE<br>8h+ media<br>90min Training	CE<br>8h+ media<br>90min Training	

Note: Temporal sequence of trial procedure. NOTV = control group, TV = intervention group, fMRI = functional magnetic resonance imaging, MDT = mechanical detection threshold, GOT = grating orientation task, TVA = theory of visual attention / visual attention testing, media = television or similar media, CE = controlled environment

Table 2

Participants Characteristics

	n (NOTV)	n (TV)	n total
Subjects recruited	40	39	79
Subjects excluded	3	2	5
Subjects included	37	37	74
Female	21	19	37
Male	16	18	37
High media consumption time[a] (30+ h/week)	6	8	14
Average media consumption time[a] (15-30 h/week)	20	14	34
Low media consumption time[a] (0-14,9 h/week)	11	15	26
Left-handed	2	4	6
Smoker	5	7	12

	mean	SD
Age, y	22.89 (22.31-23.47)	2.49
Age (NOTV)	23.46 (22.59-24.33)	2.60
Age (TV)	22.32 (21.57-23.08)	2.27
Age (Female)	22.90 (22.03-23.77)	2.72
Age (Male)	22.88 (22.10-23.66)	2.24
Total media consumption time (hours/week)	31.02 (27.81-34.23)	13.84
Specific Media consumption time[b] (hours/week)	20.29 (17.90-22.68)	10.31
Specific media consumption time[b] (NOTV, hours/week)	19.51 (16.48-22.55)	9.10
Specific media consumption time[b] (TV, hours/week)	21.07 (17.25-24.89)	11.46

Note: General information about the participating subjects. [a] Regarding specific media consumption time. [b] Specific media consumption time means passively and auditive-visually consumed electronic media with moving images (e.g., television, streaming movies via internet). Total media consumption time represents total screen time (including work on the PC or smartphone).

Table 3

Acquisition Parameters of fMRI

MP-RAGE T1 sequence parameters		Resting-state fMRI sequence parameters	
Repetition time (TR)	2300ms	Repetition time (TR)	1950ms
Echo time (TE)	2.07ms	Echo time (TE)	33.60ms
Flip angle (FA)	alpha 9°	Flip angle (FA)	90
Voxel size	1x1x1mm^3	Voxel size	1.4x1.4x1.4mm^3
In-plane field of view (FOV)	256mm	In-plane field of view (FOV)	256mm
Number of (contiguous axial) slices	192	Number of slices	100
Acquisition time	5:21min	Acquisition time	9:28min
		Multiband acceleration factor	4
**Task-related fMRI sequence parameters**			
Repetition time (TR)	484ms		
Echo time (TE)	30ms		
Flip angle (FA)	90		
Voxel size	2.5x2.5x1.5mm^3		
Number of slices	56		
Acquisition time	12 min		
Multiband acceleration factor	8		

Note: Acquisition Parameters of anatomical and functional MRI data.

Table 4

Domain	Yeo 17N	Brodmann-Area[a]
Visual	Visual 1 (VIS1)	18, 19
	Visual 2 (VIS2)	17
Somatomotor	Motor 1 (MOT1)	1, 2, 3, 4, 5, 6, 41, 42, 43
	Motor 2 (MOT2)	1, 2, 3, 4, 6, 41, 42, 43
	Motor 3 (MOT3)	22, 39, 41, 42
Attention	Dorsal attention network 1 (DAN1)	6, 7
	Dorsal attention network 2 (DAN2)	37, 39
	Including FEF and IPS	
Salience	Ventral attention network 1 (VAN1)	13, 24, 32, 39, 40
	Partly including DLPFC	
Control	Frontoparietal network 1 (FP1)	9, 10, 24, 32
	Frontoparietal network 2 (FP2)	8, 9, 10, 11, 21, 32, 46
	Frontoparietal network 3 (FP3)	40, 44, 45, 46
	Frontoparietal network 4 (FP4)	8, 9, 44, 46
	Including DLPFC (BA8, 9, 46)	
Default	Default mode network 1 (DMN1)	7, 23, 24, 31, 32
	Default mode network 2 (DMN2)	8, 9, 17, 21, 22, 39
	Default mode network 3 (DMN3)	20, 30
	Including PPC (BA7, 23, 31, 39, 40)	

Note: Resting-State Functional Networks (Yeo 17N[28]) with Spatial Cortical Classification following Brodmann[104]. FEF = frontal exe field, IPS = intraparietal sulcus, PPC = posterior parietal cortex, DLPFC = dorsolateral prefrontal cortex. [a]Overlap of Brodman-Areas between different networks is since different areas share functions, and all areas show multiple domains of activity.

Table 5

Touch-Typing Performance at Baseline

Group	n included	n excluded	Dictation mean cec/m ±SD	Training session 1 mean cec/m ±SD
NOTV	37	0	206.5 ±58.21	45.0 ±18.46
TV	37	0	208.5 ±65.79	42.5 ±14.51

Note: Dictation and Training session 1 performance shown as mean correctly entered characters per minute (cec/m) in a 10-minutes-dictation. There was no significant difference between the two groups at baseline regarding their touch-typing skills, $p > .05$.

Table 6

Touch-Typing Performance Increase - $Gain_{L3}$

	Group	$Gain_{L3}$ mean	$Gain_{L3}$ difference	$Gain_{L3}$ ±SD
Day 1 to 2	NOTV	1.34		±0.21
	TV	1.48	+0.14	±0.20
Day 1 to 3	NOTV	1.46		±0.26
	TV	1.59	+0.13	±0.24
Day 1 to 4	NOTV	1.57		±0.33
	TV	1.72	+0.15	±0.30
Day 1 to 5	NOTV	1.63		±0.38
	TV	1.79	+0.16	±0.31

Note: $Gain_{L3}$ is a marker for relative touch-typing performance gain with a predefined marker lesson (Lesson 3) as a repeating data point.

Table 7

Change in Functional Connectivity in RSN of NOTV vs TV / Pre vs Post

Network 1		Network 2		Statistical analysis
Yeo 17N	Area	Yeo 17N	Area	
VAN1	Right precentral	DMN1	Right medial prefrontal	*t* = 4.65, *p*-FDR = .002, *beta* = .19
VIS-A	Right extrastriate	DMN1	Left precuneus posterior cingulate	*t* = 3.67, *p*-FDR = .033, *beta* = .19
VIS-A	Left extrastriate	DMN1	Left precuneus posterior cingulate	*t* = 3.64, *p*-FDR = .033, *beta* = .20
VIS-A	Left extrastriate	DMN1	Right inferior parietal lobule	*t* = 3.72, *p*-FDR = .024, *beta* = .18
VIS-A	Left extrastriate	DMN1	Right precuneus posterior cingulate	*t* = 3.62, *p*-FDR = .024, *beta* = .20
VIS-A	Left extrastriate	DMN1	Right temporal	*t* = 3.23, *p*-FDR = .040, *beta* = .15
VIS-A	Left extrastriate	DMN1	Right dorsal prefrontal	*t* = 3.14, *p*-FDR = .044, *beta* = .18
VIS-A	Left extrastriate	CON-C	Right precuneus	*t* = 3.41, *p*-FDR = .027, *beta* = .18

Note: Statistical parameters of significant changes in functional connectivity between RSN. For evaluation of the change in functional connectivity the change from initial to final assessment was compared.

Table 8

Mechanical Detection Threshold (MDT) Results

Group	n included	n excluded	MDT day 0 (mN)	MDT day 7 (mN)	MDT change (mN)
NOTV	37	0	0.63 ± 0.059	0.70 ± 0.087	0.075 ± 0.10
TV	35	2	0.60 ± 0.079	0.50 ± 0.059	-0.10 ± 0.084

Note: MDT results denoted by mean ± standard error in mN.

Table 9

Grating Orientation Task (GOT) Results

Group	n included	n excluded	GOT day 0 (g_{75})	GOT day 7 (g_{75})	GOT change (g_{75})
NOTV	37	0	2.09	1.92	-0.17
TV	35	2	1.82	1.78	-0.05

Note: GOT results denoted by g_{75} and GOT change (g_{75} day 7 . g_{75} day 1).

Figures

Fig. 8

Increase in Touch-Typing Performance – $Gain_{L3}$

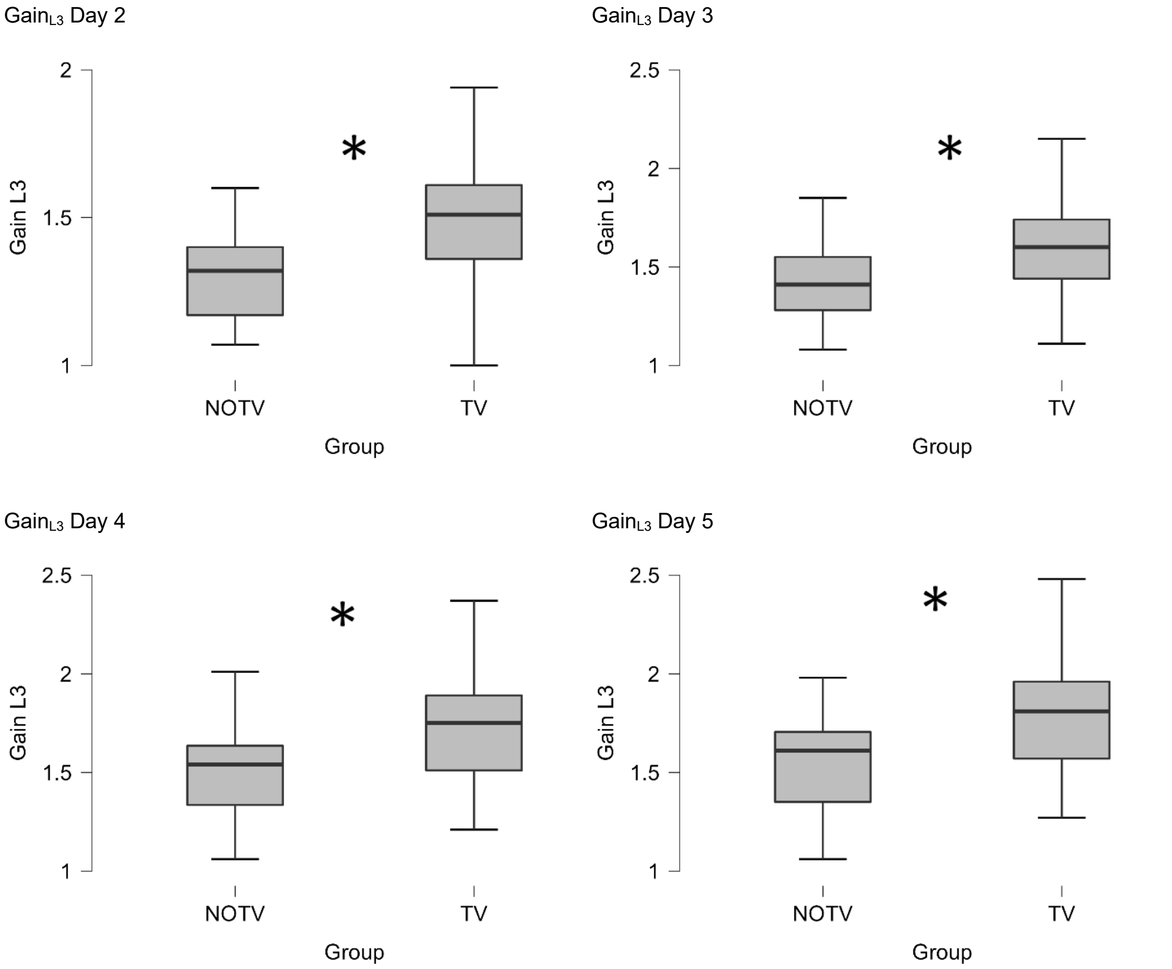

Note: Performance increase in correct entered characters in the reoccurring marker lesson in touch typing training. The difference in $Gain_{L3}$ was significant between both groups on all days of the trial, $p < .05$. Star symbol (*) is representing a significant difference between the groups. TV showed a higher $Gain_{L3}$ on all days. $Gain_{L3}$ on day 5 was 13.76% higher in TV, mean difference: .217, 95%-CI[-.36, .07], $t(70) = -3.00$, $p = .004$, $d = -.708$. Asterisk symbol (*) markes statistically significant differences between the groups.
